# Malformation of the Heart

**Published:** 1849

**Authors:** John Jackson

**Affiliations:** Goshen Indiana


### ARTICLE III.

*Case of Malformation of the Heart. Read before the Union Medical Society of Northern Indiana.* By JOHN JACKSON, M. D. of Goshen Indiana.

Oct. 30th, 1847, I attended the wife of Mr. Charles Darrow, of this town, when delivered of an apparently sound and healthy female child. It continued to thrive and grow fat without the least perceptible indisposition, until about two months old, when for the first time I was called to see it. I remarked nothing peculiar in the symptoms. Made a prescription, and on the following day it was much better.

In about two weeks afterwards the father of the child called, and said it was again affected somewhat as before and requested me to give him some medecine for it, like that previously prescribed.

Again, in about two or three we: ks, he called for more of the same medicine; for, says he, the same indisposition occasionally evinces itself, being principally a cough, and starting in its sleep.

I do not recollect any of these prescriptions, it is probable no two of them were alike.

I did not see the child from the first time I prescribed for it, until the 22d Febuary, when I was again requested to visit it. I found it with a quick pulse, slight fever, and much troubled with a cough, rather spasmodic in character. The cough, I was informed, had annoyed it for the last two months, so much as to prevent it from sleeping for more than fifteen or twenty minutes at a time. Otherwise, I remarked but little derangement. Prescribed for it, and on the following day it was much better.

Upon my visit this day, I, for the first time, observed an interuption or cessation of the action of the lungs, and of course that of the diaphragm and inter costal muscles &c. The cessation occurred regularly every 8th or 9th respiration, and lasted about 10 seconds. For more than a week there was no alteration in the symptoms evinced by the lungs, except that the cessations became more frequent and lasted longer.

For another week I continued my visits. I will here remark that from the beginning of the child's illness until the close, all its functions responded well to the prescriptions, (except those of the lungs,) and still the infant was no better, but rather worse, for it was growing weaker, and the cessation of the action of the lungs and cough, were becoming more frequent, and of course the child was more restless. At times the cough would be so convulsive as to lead me to suppose it might be the whooping cough. However, I became satisfied there was some derangement of which I was ignorant, and suggested the propriety of council. The friends called Dr. Latta to advise. After I had given him the history of the case and treatment, I mentioned to him my suspicion of some mal-formation of the heart, he allowed the probability, but considered the nerves of respiration also at fault, and suggested the use of strychnine. We accordingly prescribed it for three successive days in as large doses as, in our judgment, it could bear.

When the strychnine was first administered we thought it benefitted the child, for the coughing was not so frequent nor so convulsive. But afterwards we thought it made the infant otherwise worse for it disturbed its sleep and other functions. From this time it rapidly declined, and, on the 17th of March, died. Towards the last week the cessation of breathing would be every third or fourth respiration, and it continued about half a minute.

I should have remarked that for the last two weeks its head seemed to be much affected.

*Post Mortem examination twelve hours after death.*—Loss of flesh not very considerable. All the organs in the abdominal and thoracic cavities, except the heart presented a normalappearance. But little fat, and no serous fluid in either cavity.

We removed the heart, and npon cutting into the right auicle, discovered in the interior a pendulous white fatty looking substance of about a quarter of an inch long, half an inch in width, and a line. in thickness

The foramne ovale was open, other parts normal.

The ductus arteriosus not examined.

A small child of the same parents, died prior to the above case, having the same symptoms, but there was no post mortem examination

